# *Abcb1a* and *Abcb1b* genes function differentially in blood–testis barrier dynamics in the rat

**DOI:** 10.1038/cddis.2017.435

**Published:** 2017-09-07

**Authors:** Linlin Su, Yan C Cheng, Will M Lee, Min Zhang, Fangfang Yang, Bin Zhao, Daishu Han, Yixun Liu, Dahai Hu

**Affiliations:** 1 Department of Burns and Cutaneous Surgery, Xijing Hospital, the Fourth Military Medical University, Xi’an, Shaanxi, China; 2 The Mary M. Wohlford Laboratory for Male Contraceptive Research, Population Council, Center for Biomedical Research, New York, NY, USA; 3 School of Biological Sciences, University of Hong Kong, Hong Kong, China; 4 Department of Cell Biology, Institute of Basic Medical Sciences, Chinese Academy of Medical Sciences, Peking Union Medical College, Beijing, China; 5 State Key Laboratory of Stem Cells and Reproductive Biology, Institute of Zoology, Chinese Academy of Sciences, Beijing, China

## Abstract

During spermatogenesis, immature spermatocytes traverse the blood–testis barrier (BTB) and enter the apical apartment of seminiferous epithelium for further development. This course involves extensive junction disassembly and reassembly at the BTB. P-glycoprotein is known to be coded by two genes in rodents, namely *Abcb1a* and *Abcb1b*. Our previous studies showed that simultaneously silencing *Abcb1a* and *Abcb1b* genes in Sertoli cells impeded BTB integrity. However, the individual role of *Abcb1a* and *Abcb1b* in regulating BTB dynamics remains uninvestigated. Here, single knockdown of *Abcb1a* by RNAi impeded the *in vitro* Sertoli cell permeability barrier via redistributing TJ proteins, accelerating endocytosis, and affecting endocytic vesicle-mediated protein transportation that undermined Sertoli cell barrier. F5-peptide model was used to induce cell junction disruption and subsequent restructuring in primary Sertoli cells. F5-peptide perturbed this barrier, but its removal allowed barrier ‘resealing’. *Abcb1b* knockdown was found to inhibit barrier resealing following F5-peptide removal by suppressing the restore of the expression and distribution of junction proteins at BTB, and reducing the migration of internalized junction proteins back to Sertoli cell interface. In summary, *Abcb1a* is critical in maintaining BTB integrity, while *Abcb1b* is crucial for junction reassembly at the BTB.

At stage VIII of the seminiferous epithelial cycle in adult rat testis, preleptotene spermatocytes migrate across the blood–testis barrier (BTB) from basal into apical apartment.^1^ This course involves extensive junction disruption and restructuring at Sertoli cell–cell interface to facilitate germ cell movement.^2^ In the meanwhile, the immunological integrity of the BTB has to be maintained at all times in order to separate postmeiotic germ cell antigens from the immune system. Uncovering the underlying mechanisms that manipulate the timely ‘open’ and ‘close’ of the BTB would be able to explain the microscopical observation: a migrating spermatocyte is ‘trapped’ between a ‘disrupting’ and a ‘newly formed’ tight junction (TJ) barrier at the BTB region of Sertoli cells.^3^

In the past decade, scientists have made extensive efforts in discovering biomolecules that regulate BTB junctional complexes.^2, 4^ Among these, P-glycoprotein is critical in maintaining Sertoli cell barrier function,^5^ it is structurally associated with several putative TJ proteins at Sertoli cell BTB, such as occludin, junctional adhesion molecule-A (JAM-A), and claudin-11.^6^ P-glycoprotein is encoded by *Abcb1a* and *Abcb1b* genes in rodents. P-glycoprotein deletion by co-silencing *Abcb1a* and *Abcb1b* in Sertoli cells significantly impaired TJ barrier function, affected occludin phosphorylation by the activation of focal adhesion kinase (FAK), and disturbed the endocytosis of junctional complexes that further destabilized barrier function.^5^

*Abcb1a* and *Abcb1b* genes encode rat ABCB1A and ABCB1B proteins (two isoforms of rat P-glycoprotein), respectively, which together functionally resemble the human ABCB1 protein (namely, human P-glycoprotein).^7, 8, 9^ Although both *Abcb1a* and *Abcb1b* encode P-glycoprotein, many studies have shown that these two genes often response differentially under the same stimulation, or play different roles in certain cellular events. *Abcb1a* mRNA level was found to increase in hippocampus and liver, also presented an upward trend in the kidney of vitamin A-deficient rats by qRT-PCR analysis, whereas *Abcb1b* mRNA level was induced in hippocampus but downregulated in kidney, liver, and cerebral cortex.^10^ Researchers have also found remarkably higher *Abcb1b* gene expression, and lower *Abcb1a* gene in the post-natal day 14 rat microvessels than that in adult rat microvessels.^11^

Endocytic vesicle-mediated junctional protein recycling is reported to regulate junction restructuring to maintain barrier integrity,^12, 13, 14^ which enables the renovation of integral membrane proteins besides normal protein synthesis.^12^ The testis is likely to take a similar way to guide junction reconstruction at Sertoli cell surface since endocytosis was found to be involved in the course of spermiation at the interface of Sertoli cell and late spermatid.^15, 16^ Moreover, primary Sertoli cells could form an *in vitro* BTB that features a functional TJ permeability barrier.^17, 18, 19, 20^ Thus, this *in vitro* Sertoli cell system was employed to examine the effects of *Abcb1a* or *Abcb1b* knockdown on the kinetics of endocytosis and recycling of integral membrane proteins at the BTB.^21^

In this study, we examined the individual role of *Abcb1a* or *Abcb1b* on BTB dynamics, especially focused on disassembly and reassembly of Sertoli cell–TJ barrier by using RNAi combined with F5-peptide model, which was found to reversibly disrupt the BTB integrity both *in vivo* and *in vitro*.^22^ Interestingly, we found that the barrier dynamic is managed by the coordination of *Abcb1a* and *Abcb1b* genes, which differentially participate in BTB disassembly and reassembly by affecting the destiny of endocytosed BTB junctional proteins. Above findings thus propose a novel mechanism the testis has used to control the proper ‘on’ and ‘off’ of the BTB, and report differential roles of *Abcb1a* and *Abcb1b* in BTB homeostasis during spermatogenesis.

## Results

### Knockdown of *Abcb1a* or *Abcb1b* in Sertoli cells by RNAi differentially affects barrier function

**Figure 1:**
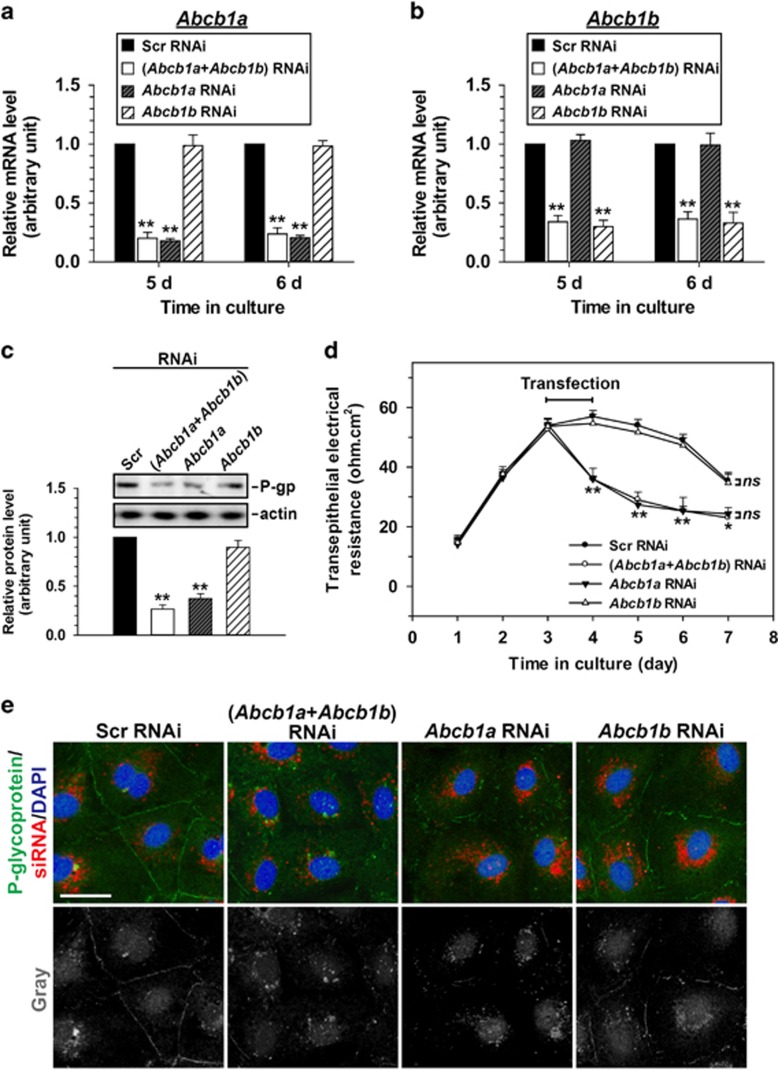
Study to assess the effect of *Abcb1a* and *Abcb1b* single or double knockdown by RNAi on Sertoli cell BTB function *in vitro*. Real-time PCR showing changes of the mRNA levels of *Abcb1a* (**a**) and *Abcb1b* (**b**) after *Abcb1a/Abcb1b* single or double knockdown by RNAi. (**c**) Representative immunoblots illustrating the change of P-glycoprotein after *Abcb1a/Abcb1b* single or double RNAi in Sertoli cells. Immunoblotting data was normalized against actin with the value in scramble (Scr) RNAi group arbitrarily set at 1. Each bar is the mean±S.D. of three independent experiments using different batches of Sertoli cells. *P<0.05; **P<0.01. (**d**) The effect of *Abcb1a* and *Abcb1b* single or double knockdown on Sertoli cell–TJ permeability barrier was monitered by TER. *ns*, no significant difference. (**e**) Sertoli cells cultured at 0.05 × 10^6^ cells/cm^2^ on Matrigel-coated coverslips were cotransfected with siGLO Red (a transfection indicator) with Scr, *Abcb1a*+*Abcb1b*, *Abcb1a* alone or *Abcb1b* alone siRNA duplexes and stained for P-glycoprotein to investigate changes in its cellular distribution. Micrographs in the lower row are the corresponding grayscale images of the true-color images in the upper row, scale bar=20 *μ*m, which applies to all micrographs

P-glycoprotein has two isoforms in rats, which are separately encoded by *Abcb1a* and *Abcb1b*.^23^ A previous study from our group has shown that simultaneous knockdown of *Abcb1a* and *Abcb1b* would induce a disruption of the BTB,^5^ however, the individual role of *Abcb1a* and *Abcb1b* in BTB integrity has not been investigated. To clarify whether *Abcb1a* and *Abcb1b* have the identical or differential role in maintaining BTB integrity, we separately silenced *Abcb1a* or *Abcb1b* in cultured Sertoli cells using siRNA duplexes specific to *Abcb1a* or *Abcb1b*. Isolated Sertoli cells were incubated in normal DMEM/F12 culture medium for 72 h, followed by 24 h transfection of scramble (negtive control, Scr), *Abcb1a*-specific, *Abcb1b*-specific or (*Abcb1a-*specific+*Abcb1b*-specific) (positive control) siRNA duplexes. Afterwards, the reaction mixture was withdrawn, Sertoli cells were washed with plain medium three times, incubated for another 1 to 2 days, and finally harvested for real-time RCR (Figures 1a and b), immunoblot (Figure 1c), trans-epithelial electrical resistance (TER) measurement (Figure 1d) and immunofluorescence analysis (Figure 1e). qPCR results revealed the efficiency of RNAi transfection, presenting an ~80% decrease in *Abcb1a* mRNA level (Figure 1a) and an ~70% decrease in *Abcb1b* mRNA level (Figure 1b). In addition, the *Abcb1a* and *Abcb1b* siRNA duplexes did not affect each other’s RNA level after transfection (Figures 1a and b), indicating the specificity of these siRNA duplexes used. At the translational level, both *Abcb1a* alone and (*Abcb1a*+*Abcb1b*) double knockdown reduced P-glycoprotein level by 65–75%. On the other hand, although knockdown of *Abcb1b* alone caused a slight decline in P-glycoprotein level, the effect was not statistically significant compared to that in Scr RNAi group (Figure 1c), indicating *Abcb1b*-encoded P-glycoprotein is a minority in rat Sertoli cells compared to *Abcb1a*-encoded P-glycoprotein. At the functional level, single knockdown of *Abcb1a* produced a similar dramatic disruptive effect on Sertoli cell–TJ barrier integrity as that when *Abcb1a* and *Abcb1b* were simultaneously silenced (Figure 1d). On the other side, lack of *Abcb1b* alone did not cause any damage on the barrier intactness compared with Scr RNAi control (Figure 1d). By immunofluorescent staining, P-glycoprotein was observed almost exclusively at cell interface in scramble siRNA-transfected Sertoli cells, while it almost disappeared at cell boundary except for a few residual stainings in the cytoplasm when *Abcb1a* alone or both *Abcb1a* and *Abcb1b* were silenced (Figure 1e). *Abcb1b* single knockdown did not change P-glycoprotein localization in Sertoli cells (Figure 1e).

### Abcb1a single knockdown in Sertoli cell epithelium facilitates the endocytosis of BTB junctional proteins and regulates the endocytosed protein trafficking

**Figure 2:**
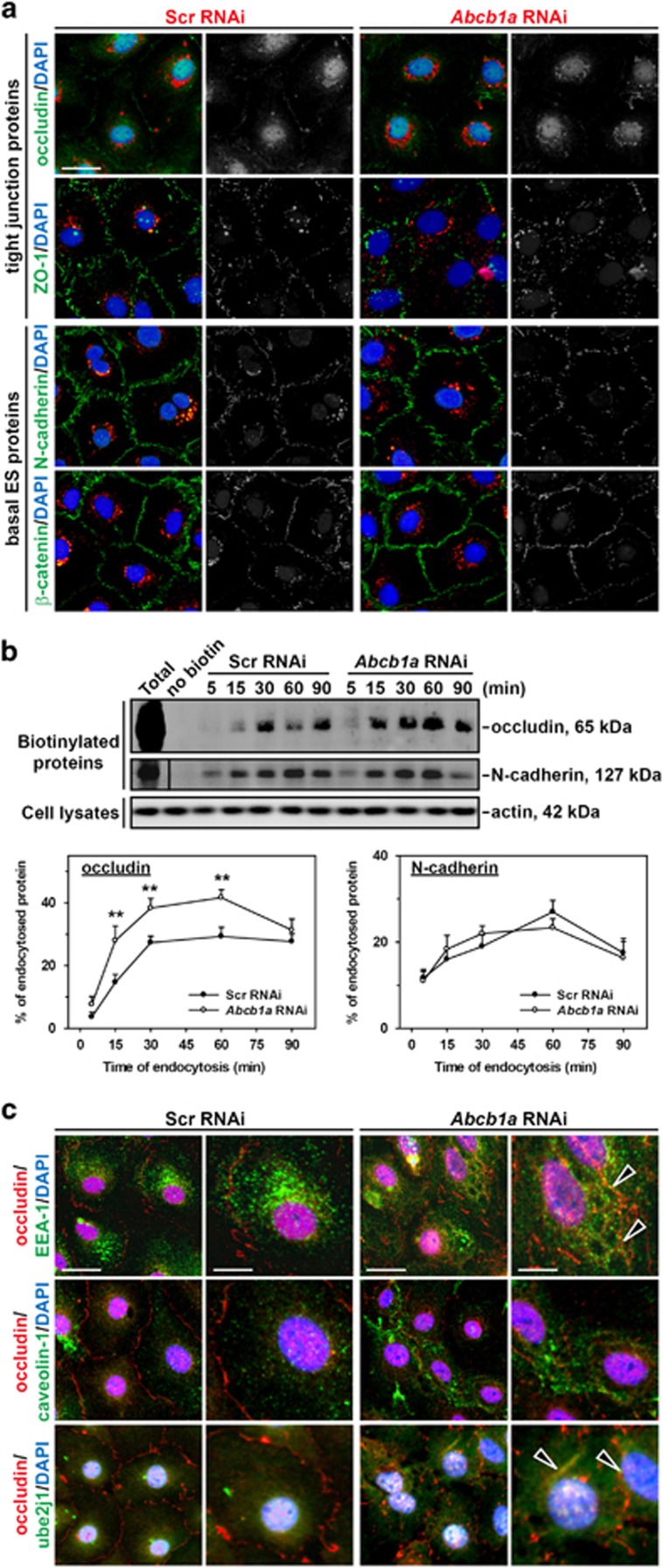
Study to assess the effect of *Abcb1a* single knockdown on the endocytosis of integral membrane proteins at the BTB and the endocytic vesicle-mediated protein trafficking in Sertoli cell epithelium. (**a**) Sertoli cells were cotransfected with siGLO Red with either scramble or *Abcb1a* siRNA duplexes, and stained for TJ proteins (e.g., occludin and ZO-1) and basal ES proteins (e.g., N-cadherin and *β*-catenin) to investigate changes in protein distribution at the cell–cell interface. Although loss of *Abcb1a* by RNAi had no apparent effect on the fluorescent intensity of BTB proteins, it induced the mislocalization of occludin and ZO-1, but not N-cadherin or *β*-catenin, at the cell–cell interface. Micrographs in the second and fourth columns are the corresponding grayscale images of the true-color images on the left in order to better depict changes in protein localization, scale bar, 20 *μ*m, which applies to all micrographs. (**b**) Two days after RNAi transfection, Sertoli cells were subjected to cell surface protein biotinylation and then incubated at 35 °C to allow endocytosis, reactions were terminated at specified time points. About 400 *μ*g of total proteins from samples at each time point was used to estimate the kinetics of protein endocytosis. The kinetics of internalization of occludin and N-cadherin were summarized in histograms. The percentage of internalized proteins *versus* total biotinylated proteins is shown on the *y* axis and plotted against time. Each data point is the mean±S.D. of results from three separate experiments, **P<0.01. (**c**) Cellular colocalization of EEA-1 (an endosome marker), caveolin-1 (a transcytosis marker) or ube2j1 (an intracellular protein degradation marker) with occludin in Sertoli cells was examined by dual-labeled immunofluorescence analysis after *Abcb1a* knockdown. White arrowheads denote an increase in the colocalization of occludin with EEA-1 and ube2j1, but not with caveolin-1, after *Abcb1a* knockdown. DAPI (blue) was used to visualize nuclei, scale bars on the left column in each treatment group, 20 *μ*m, scale bars on the right column in each treatment group, 10 *μ*m

When Sertoli cells were transfected with scramble siRNA and siGLO Red, a transfection indicator, two putative TJ markers occludin and zonula occludens-1 (ZO-1), and two basal ectoplasmic specialization (basal ES, a special form of adhension junction in the testis) marker proteins N-cadherin and *β*-catenin, were detected predominantly at Sertoli cell–cell interface (Figure 2a). After *Abcb1a* knockdown, occludin and ZO-1 lost their regular arrangement and showed extensive disruption by migrating ino cytoplasm from the membrane (Figure 2a, first two rows), while the distribution of N-cadherin and *β*-catenin did not change (Figure 2a, last two rows). These findings coincide with the results when *Abcb1a* and *Abcb1b* were simultaneously silenced^5^ and illustrate that *Abcb1a* gene regulates BTB integrity by affecting TJ protein distribution, but not basal ES proteins.

Later, we found that loss of *Abcb1a* remarkably promoted the internalization of occludin, but not N-cadherin in cultured Sertoli cells (Figure 2b), illustrating that *Abcb1a* knockdown could destroy barrier integrity via accelerating the endocytosis of TJ integral membrane proteins. The colocalization of occludin (Figure 2c)/N-cadherin (Supplementary Figure 1) with early endosome antigen-1 (EEA-1), caveolin-1, or ubiquitin-conjugating enzyme E2 J1 (Ube2j1) was assessed by dual-labeled immunofluorescent microscopy. Results showed that occludin-EEA-1 as well as occludin-Ube2j1 association was increased partly due to the increased internalization of occludin following *Abcb1a* knockdown (Figure 2c). In contrast, associations of N-cadherin with EEA-1, caveolin-1, or Ube2j1 were not affected by *Abcb1a* RNAi (Supplementary Figure 1). These findings are coincident with our earlier results when *Abcb1a* and *Abcb1b* were simultaneously silenced^5^ and establish that single knockdown of *Abcb1a* significantly promotes EEA-1-mediated TJ protein, such as occludin, internalization as well as Ube2j1-mediated degradation, respectively, thus impeding the TJ barrier function in Sertoli cells.

### F5-peptide reversibly damages the TJ barrier function in Sertoli cells

**Figure 3:**
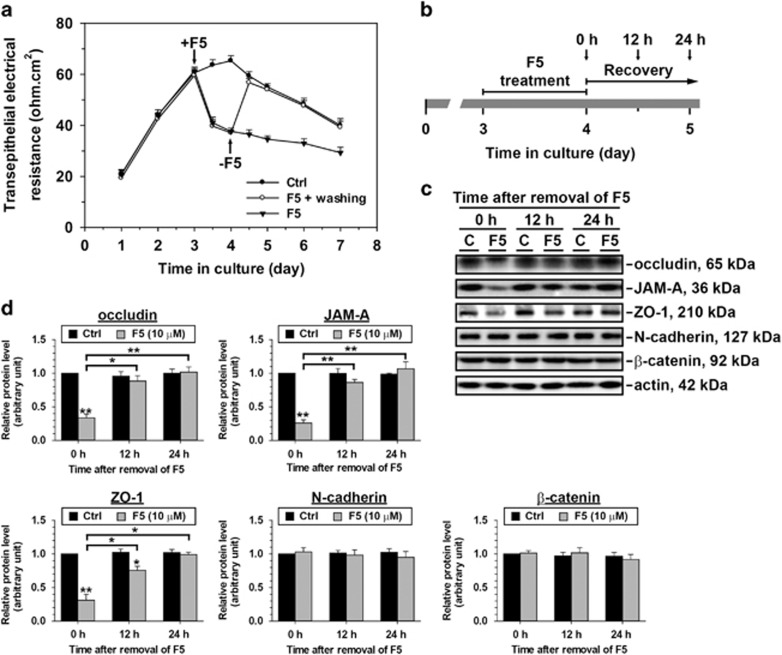
F5-peptide causes a transient disruption of the BTB integrity and changes in the steady-state levels of proteins at the BTB in primary Sertoli cell cultures. (**a**) In TER experiment, Sertoli cells were treated with vehicle control (normal culture medium, Ctrl) or synthetic F5-peptide (10 *μ*M) on day 3 of culture. F5-peptide-treated Sertoli cells displayed a drastic reduction of the TJ permeability barrier from 12 h post-treatment onward when compared with Ctrl. In one treatment group, F5-peptide was removed from the Sertoli cell culture after 24 h treatment (that is, on day 4 of culture) to allow the ‘resealing’ of the disrupted TJ barrier. A representative dataset is shown (*n*=4). (**b–d**) Sertoli cells were treated with Ctrl or F5-peptide for 24 h. In F5-peptide treatment group, F5-peptide was removed after 24 h and cells were rinsed and replenished with normal culture medium. Cell lysates were then collected 0, 12 and 24 h after F5-peptide removal to analyze changes in the protein levels of various junction markers at the BTB. The level of cytoskeletal protein actin serves as loading control. Representative immunoblots are shown in *C*, where *n*=3–5. The densitometry results are summarized in *D*. The F5-peptide-treated group from each time point was normalized against corresponding actin and then compared with the Ctrl group at the same time point which was arbitrarily set at 1. Protein levels in the F5-peptide-treated group were also compared between different time points to assess the degree of protein recovery. The disruptive effect of F5-peptide on junction protein level was observed for integral membrane proteins of TJ (e.g., occludin and JAM-A) and basal ES (e.g., N-cadherin), and their corresponding adaptors (e.g., ZO-1 and *β*-catenin). TJ proteins (occludin, JAM-A and ZO-1) show a significant rebound from the lowered protein levels after F5-peptide removal, but not basal ES proteins (N-cadherin and *β*-catenin). These data thus illustrate the reversible effect of F5-peptide on junction integrity and junction protein levels. *P<0.05; **P<0.01; statistical difference was analyzed by ANOVA with a *post hoc* Tukey/Kramer test

Our previous work has reported that the synthetic F5-peptide could reversibly disintegrate Sertoli cell barrier,^22^ similar results are presented in Figure 3a. Nevertheless, whether the restore of BTB structural proteins holds pace with the ‘resealing’ of the impaired Sertoli cell barrier is still unclear. In order to answer this question, we measured the steady-state protein levels of several selected BTB markers at specific time points after F5-peptide removal during the course of barrier resealing. Figure 3b shows the regimen, briefly, cells were treated with 10 *μ*M synthetic F5-peptide for 24 h to induce barrier disruption or cultured in nomal medium (vehicle control) for the same period. Thereafter, cells were washed and cultured in fresh medium to allow junction recovery, which was designated as time 0. Cell lysates were collected at 0, 12 and 24 h to measure the protein level changes. A decline in barrier integrity (Figure 3a) and the downregulated levels of TJ proteins (e.g., occludin, JAM-A and ZO-1; Figures 3c and d) were noticed right after F5-peptide removal (0 h). At 12 h after F5-peptide removal, the junction integrity recovered and there was no difference between control and experiment groups (Figure 3a), the declined TJ protein levels were also upregulated (Figures 3c and d). 24 h later, all three TJ protein levels had recovered to the corresponding control levels (Figures 3c and d). Notably, the protein levels of N-cadherin and *β*-catenin, two basal ES markers, showed no difference between F5-peptide treated group and control group following removal of F5-peptide.

**Figure 4:**
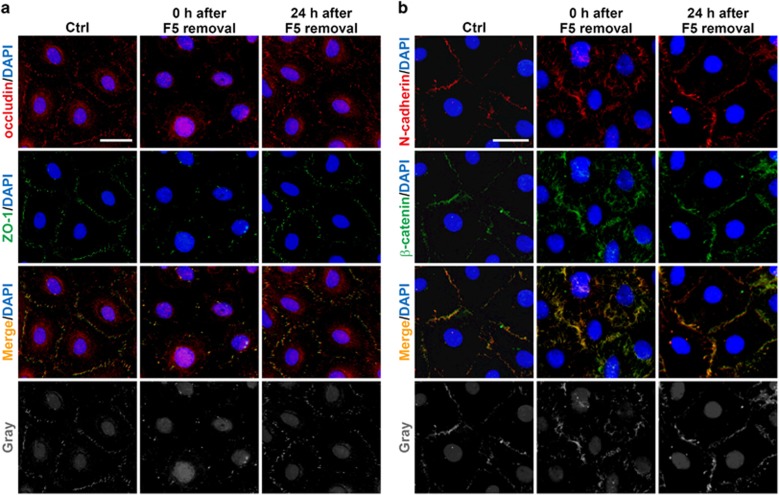
Changes in the distribution of junction proteins following treatment of Sertoli cells with F5-peptide. The distribution of occludin/ZO-1 (**a**) and N-cadherin/*β*-catenin (**b**) in Sertoli cells was studied after the treatment and removal of F5-peptide. At 0 h after F5-peptide removal, fewer occludin/ZO-1 was observed at the cell–cell interface, and N-cadherin/*β*-catenin was shown to move away from the cell border into cytoplasm. While at 24 h after F5-peptide removal, reappearance of these junction proteins was observed at the cell–cell interface. These data again illustrate the reversibility of junction disruption induced by F5-peptide. Micrographs in the fourth rows in **a** and **b** are the corresponding grayscale images of the true-color images in the third rows, in order to better depict changes in protein localization, scale bars=20 *μ*m, which applies to all micrographs

Next we performed immunofluorescence staining to further confirm above findings (Figure 4). In the control group, occludin and ZO-1 (Figure 4a), as well as N-cadherin and *β*-catenin (Figure 4b), were detected and colocalized. At 0 h after removing F5-peptide, considerably fewer staining of occludin and ZO-1 remained at cell interface (Figure 4a), although N-cadherin and *β*-catenin did not show an apparent decline in fluorescence intensity, their localization markedly moved away from cell boundary into cytosol (Figure 4b). At 24 h after F5-peptide was removed, the staining of occludin and ZO-1 reappeared (Figure 4a), N-cadherin and *β*-catenin also relocalized back (Figure 4b), suggesting a restoration from F5-peptide-induced barrier damage.

**Figure 5:**
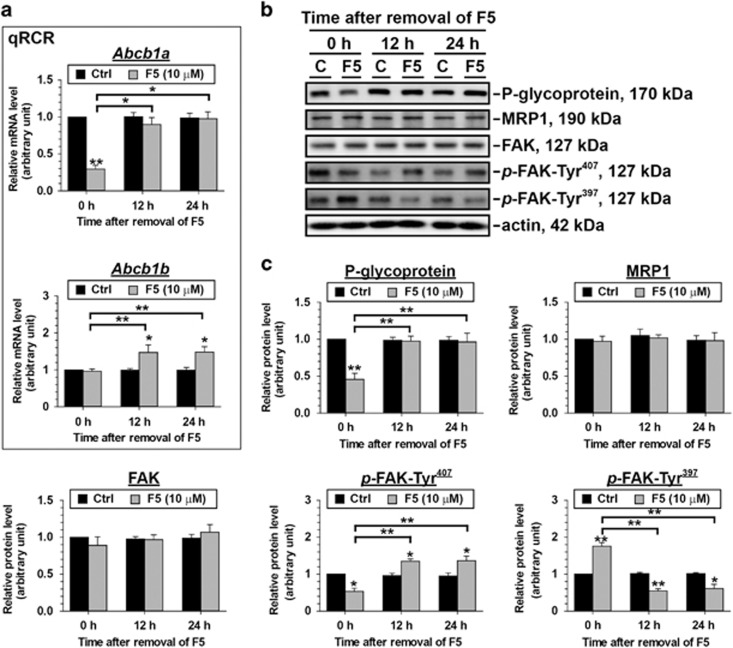
Changes in the steady-state mRNA levels of *Abcb1a* and *Abcb1b* and protein levels of FAKs following treatment of Sertoli cells with F5-peptide and its removal. Sertoli cells cultured at 0.5 × 10^6^ cells/cm^2^ on Matrigel-coated multiwell plates were treated with F5-peptide as depicted in Figure 3b. *Abcb1a* and P-glycoprotein showed a drastic reduction in the mRNA or protein level after 24 h-F5-peptide treatment, respectively, followed by a significant recovery at 12 h after F5-peptide removal. The mRNA level of *Abcb1b* was not affected by F5-peptide but increased after its removal. The protein level of MRP1 and FAK remained unchanged during and after F5-peptide treatment. The *p*-FAK-Tyr^407^ and *p*-FAK-Tyr^397^ involved FAK signaling pathway was antagonistically inhibited or induced during F5-peptide treatment and after its removal. qPCR results and representative immunoblots are shown in (**a**) and (**b**), respectively, where *n*=3–4. The densitometry of the proteins is analyzed and summarized in (**c**). *P<0.05; **P<0.01; statistical difference from the Ctrl group was analyzed by ANOVA with a *post hoc* Tukey/Kramer test

Interestingly, F5-peptide only caused the decline in mRNA level of *Abcb1a* but not *Abcb1b* (time 0 h column, Figure 5a), also, *Abcb1a* mRNA level was recovered after F5-peptide removal (Figure 5a). The effect of F5-peptide on P-gp level displayed the similar trend as that on *Abcb1a* (Figures 5b and c). The protein level of MRP1, another major drug transporter in the rat testis, on the other hand was not affected by F5-peptide treatment (Figures 5b and c). This illustrates differential responses of drug transporters toward F5-peptide and may indicate differential roles of drug transporters on regulating BTB dynamics. Importantly, FAK signaling was activated during F5-peptide treatment although total FAK protein level in F5-peptide treatment group remained the same as that in the control group during the first 24 h of Sertoli cell–TJ barrier disruption, a decline and a surge in its phosphrylated forms *p*-FAK-Tyr^407^ and *p*-FAK-Tyr^397^ were observed after 24 h-F5-peptide treatment (that is, 0 h after romoval of F5), respectively (Figures 5b and c). FAK signaling pathway was also involved in Sertoli cell–TJ barrier re-establishment, showing elevated *p*-FAK-Tyr^407^ level and downregulated *p*-FAK-Tyr^397^ level after F5-peptide removal when compared to corresponding controls (Figures 5b and c). Above findings illustrate the FAK signaling does not only participate in junction disassembly caused by F5-peptide, but also junction recovery following F5-peptide withdrawal.

### Knockdown of Abcb1b alone impedes TJ barrier recovery and abolishes the recycling of internalized withdrawal withdrawal BTB integral membrane proteins

**Figure 6:**
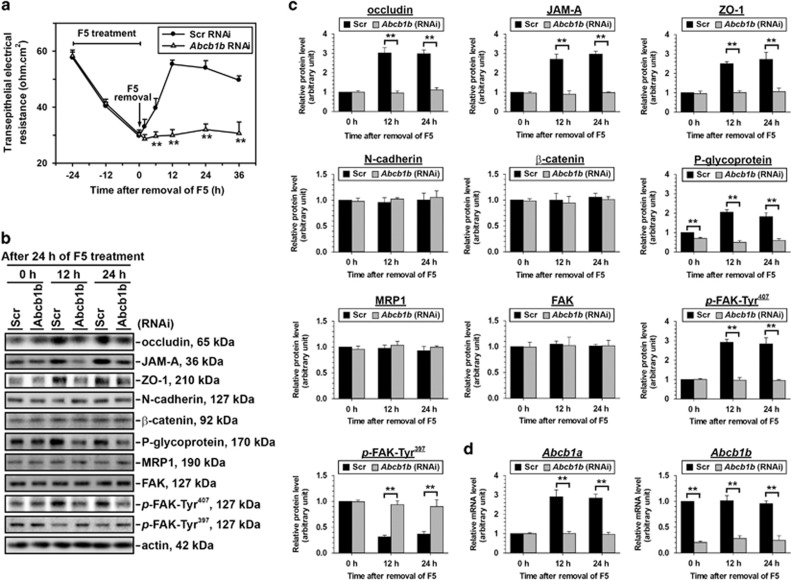
A study using the F5-peptide model to examine the role of *Abcb1b* in TJ permeability barrier reassembly in Sertoli cell epithelium. 1.5 days after transfection with Scr or *Abcb1b* siRNA duplexes, Sertoli cells were treated with 10 *μ*M synthetic F5-peptide for 24 h to induce junction restructuring. (**a**) Upon F5 removal, the resealing of the disrupted TJ permeability barrier only occurred in cells transfected with scramble siRNA duplexes, TJ barrier integrity in cells with *Abcb1b* knockdown showed permanent disruption. (**b**,**c**) Levels of various TJ and basal ES structural and signal proteins were investigated during junction restructuring induced by F5-peptide in Scr or *Abcb1b* RNAi transfected Sertoli cells. Immunoblots were representative of at least three independent experiments, statistical difference was analyzed by one-way ANOVA along with the Tukey/Kramer test, **P<0.01. (**d**) qPCR results showed changes in the mRNA levels of *Abcb1a* and *Abcb1b* during junction restructuring induced by F5-peptide after RNAi transfection

**Figure 7:**
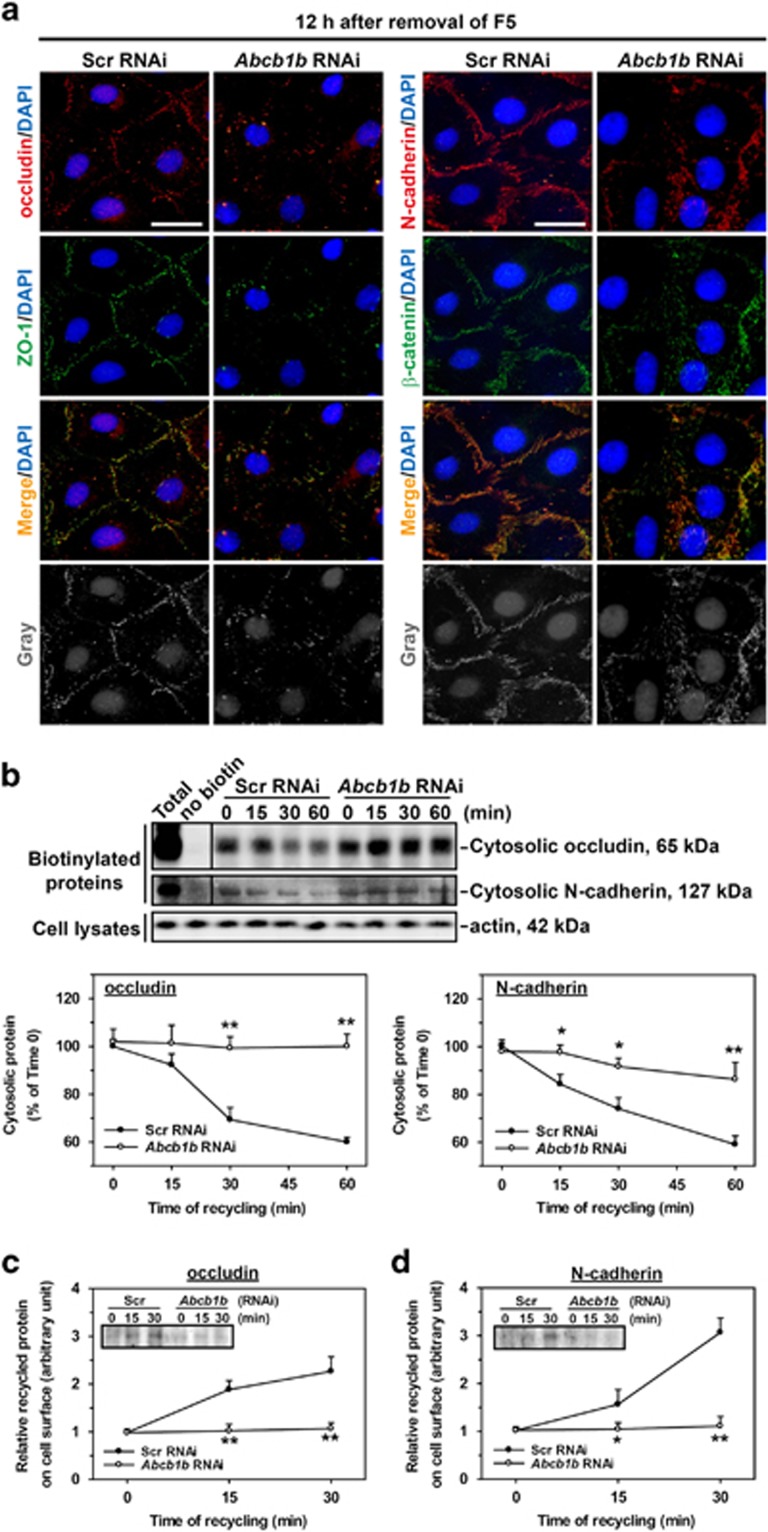
A study to assess the effect of *Abcb1b* single knockdown on the fate of endocytosed BTB integral membrane proteins induced by F5-peptide in Sertoli cell epithelium. (**a**) Changes in the distribution of proteins at the Sertoli–Sertoli cell interface were examined by immunofluorescence microscopy. Junction proteins, including occludin, ZO-1, N-cadherin and *β*-catenin, relocalized back to the cell surface at 12 h after F5-peptide removal in the scramble silencing group. However, cells with *Abcb1b* knockdown displayed a mislocalization of these junction proteins. Micrographs in the fourth row are the corresponding grayscale images of the true-color images in the third row to better depict changes in protein localization, scale bars=20 *μ*m, which applies to all micrographs. (**b**–**d**) Recycling assay illustrated the fate of the internalized BTB integral membrane proteins induced by F5-peptide in Src or *Abcb1b* RNAi treated Sertoli cells. After biotinylation of cell surface proteins, Sertoli cells were incubated at 35 °C for 2 h to allow endocytosis. Biotin on the uninternalized cell surface proteins was stripped. Cells were then incubated at 35 °C for various time points to allow recycling of internalized proteins back to the cell surface. The newly appeared biotinylated (and recycled) proteins on the cell surface were obtained by 0.01% trypsin, whereas proteins remaining in the cytosol were collected by IP buffer. (**b**) Immunoblots and percentage of internalized and biotinylated proteins remaining in the cytosol over time in the recycling assay were shown. The percentage of internalized and biotinylated proteins at time 0 was arbitrarily set at 100. Each bar is the mean±S.D. of three independent experiments using different batches of primary Sertoli cell cultures. In each experiment, each time point had triplicate cultures. (**c**,**d**) Reappearance of internalized and biotinylated occludin and N-cadherin on the Sertoli cell surface after Scr or *Abcb1b* RNAi was monitored by immunoblotting (the insets) in three independent experiments with triplicate cultures for each time point in each experiment. The level of protein at time 0 was arbitrarily set at 1. Statistical analysis was performed by two-way ANOVA followed by Dunnett’s test, comparing between *Abcb1b* RNAi group *versus* its corresponding Scr RNAi group and over time. *P<0.05; **P<0.01

Sertoli cells were transfected with non-targeting control or *Abcb1b*-specific siRNA for 1.5-day and then treated with 10 *μ*M F5-peptide for additional 24 h. Following the removal of F5-peptide, the disrupted TJ junction began to reseal in control group, but not in *Abcb1b* knockdown group (Figure 6a). It was noted that the downregulated protein levels of occludin, JAM-A and ZO-1surged dramatically after F5-peptide removal in control group while remained lower in *Abcb1b* RNAi group (Figures 6b and c). The protein levels of N-cadherin and *β*-catenin showed no difference between the control and *Abcb1b* RNAi group during the whole recovery process monitered (Figures 6b and c). The *Abcb1b* mRNA level reduced by 70–80% by *Abcb1b*-specific RNAi and remained at lower level during 24-h junction recovery course (Figure 6d), while *Abcb1b* RNAi did not alter *Abcb1a* mRNA level, indicating the specificity of the *Abcb1b* siRNA duplex used (Figure 6d). During the junction resealing process in scramble RNAi group, the mRNA level of *Abcb1a* (Figure 6d) and the protein level of P-glycoprotein notably rebounded (Figures 6b and c), however, the total P-glycoprotein level significantly decreased in *Abcb1b-*silenced group (Figures 6b and c). In addition, although the total FAK protein level kept unchanged during the junction re-establishment, *p*-FAK-Tyr^407^ and *p*-FAK-Tyr^397^ were significantly inhibited or activated in *Abcb1b* knockdown group, respectively (Figures 6b and c). In control cells, the immunofluorescent staining of occludin, ZO-1, N-cadherin, and *β*-catenin was observed to relocalize back to cell–cell interface at 12 h after F5-peptide withdrawal. While a disturbed relocalization of junction markers was noticed in cells transfected with *Abcb1b* RNAi (Figure 7a). Above findings thus suggest that the downregulation of *Abcb1b* could impede the restoration of a damaged TJ barrier caused by F5-peptide.

To confirm above data, the endocytosis and recycling of two selected junctional proteins after scramble or *Abcb1b* RNAi treatment were assessed. It is known that part of the internalized BTB proteins would gradually relocate back to cell surface. The dynamics on the descending of endocytosed proteins in cytoplasm (Figure 7b) and the recurrence of the internalized proteins onto cell surface (Figures 7c and d) were thus quantified, for which the technical principles can be found in an early report.^21^ The vanishment of cytosolic internalized occludin and N-cadherin was inhibited in *Abcb1b*-silenced group (Figure 7b). On the other hand, the elevated level of recurrence of endocytosed occludin and N-cadherin was observed in control cells, while no sign of reappearance of these proteins on the cell surface was seen in *Abcb1b*-silenced group (Figures 7c and d). These results suggest that *Abcb1b* knockdown destroyed the recycling dynamics of BTB proteins.

## Discussion

In rodents, *Abcb1a* and *Abcb1b* genes encode ABCB1A and ABCB1B isoforms of P-glycoprotein, respectively,^23, 24^ they possess specific substrates and/or tissue distribution while sometimes overlapped.^25, 26^ For example, the *Abcb1b* mRNA level in placenta exhibited an obvious relevance with the progesterone concentration in maternal plasma, but not *Abcb1a*.^27^ *Abcb1a* mRNA level in fetal brain increases with advancing gestation, while *Abcb1b* mRNA level remains low.^28^ Yet the regulatory control of P-glycoprotein function and activity in the testes is largely unknown, probably *Abcb1a* and *Abcb1b* are separately regulated. Our present study has revealed the differential participation of *Abcb1a* and *Abcb1b* in BTB dynamics.

*Abcb1a* and *Abcb1b* are differentially regulated depending on the promoter region in different organs/tissues. For instance, *Abcb1a* prefers to express in many tissue barriers such as the blood-brain barrier, blood–testis barrier compared to *Abcb1b*,^24, 29^ while *Abcb1b* highly exists in placenta and ovaries. Studies also indicated that the epression of *Abcb1a* was closely related with the activity of P-glycoprotein.^11^ In the current study, the result that *Abcb1b* knockdown-induced decrease on P-glycoprotein level was not as severe as *Abcb1a* knockdown-induced P-glycoprotein level downregulation may suggest that *Abcb1a* is the pridominant isoform in rat Sertoli cells which is in concert with previous report.

*Abcb1a* is known to be predominantly expressed by rat Sertoli cells compared to *Abcb1b*.^30, 31^ In this study, the disruption and restructuring of BTB were found to be mediated by coordination of *Abcb1a* and *Abcb1b*. Single suppression of *Abcb1a* impeded the barrier function in cultured Sertoli cells by affecting TJ protein distribution, accelerating protein endocytosis, and interfering the fate of endocytosed proteins at the BTB (Figure 2), which is in consistance with the results when *Abcb1a* and *Abcb1b* genes were simultaneously silenced.^5^ On the other hand, silencing *Abcb1b* in Sertoli cells remarkably inhibited barrier resealing following F5-peptide removal by suppressing the restore of the expression and distribution of BTB junction proteins, also reducing the recycling of internalized biotinylated proteins (Figures 6 and 7). It is noted that previous studies have found *Abcb1a* to be the predominant form in rat BTB while *Abcb1b* is a more inducible isoform, these results are similar to ours since *Abcb1b* was required for barrier recovery after disruption.

Isoform regulation of the BTB was also observed in an early study. Pelletier RM *et.al.* found that the distribution of alpha^+^ and alpha^−^ isoforms of TJ adaptor protein ZO-1 in guinea pig testis was different at the site of Sertoli cell–TJ that is responsible for the blood–testis barrier.^32^ Also, alpha^+^ and alpha^−^ were predominantly expressed during puberty and adulthood, respectively, indicating that alpha^+^ was predominant in the period of extensive junction assembly/disassembly at the BTB.^32^ Moreover, since TJ is an actin-based cell junction type, a correspondence between ZO-1 alpha^+^ and F-actin may indicate the influence of the spatial organizaiton of subsurface actin on alpha^+^ or alpha^−^-involved TJ assembly/disassembly.^32^ In human, the isoform-specific activities of protein phosphatase 1 (PP1) in heart failure and atrial fibrillation have been reported,^33^ results proposed that isoform-specific targeting of PP1*α* activity might be an innovative stragegy for human cardiac diseases but not PP1*β* or PP1*γ*. ^33^

Taken together, the current study suggests a novel mechanism by which the timely disassembly and reassembly of the BTB junctions are well coordinated to allow the proper migration of developing spermatocytes, also reports the differential roles of *Abcb1a* and *Abcb1b* in the BTB homeostasis during spermatogenesis in rat testis.

## Materials and methods

### Animals

Animal experiments involved in this study were authorized by the Institutional Animal Use and Care Committee of the Fourth Military Medical University (Xi'an, China). All male pup Sprague-Dawley (SD) rats at 20-day-old were purchased from the Animal Center. Experiments were executed in concert with the Guide for the Care and Use of Laboratory Animals issued by the National Research Council. Rats had free access to water and standard rat chow, and were maintained in room with 12-h light/12-h dark cycle.

### Sertoli cell culture

Sertoli cells were obtained from the 20-day-old SD rat testes, they were fully differentiated without further division. Cells were then cultured on Matrigel-coated plates at different densities of 1.2 × 10^6^ cells/cm^2^, 0.5 × 10^6^ cells/cm^2^ or 0.05 × 10^6^ cells/cm^2^ depending on different usage. The culture medium was serum-free F12/DMEM containing several growth factors and antibiotics. 48 h after plating, cell culture received hypotonic treatment to remove remaining germ cells to reach a >98% Sertoli cell purity. Thereafter, Sertoli cells were collected at different time points for different experiments.

### Quantitative real-time PCR

Total RNA was extracted from homogenized cells using Total RNA Isolation Kit (Takara, Japan). The purity of obtained RNA was determined by A260/A280 ratio. 2.0 *μ*g of RNA sample was reversely transcribed with PrimeScript RT Reagent Kit (Takara, Japan). The resulting cDNA was then amplified by using SYBR Premix Ex Taq Kit (Takara, Japan) with primer pairs specific to target genes as shown in Supplementary Table 1, which were finally normalized against the transcriptional level of housekeeping gene GAPDH. The thermal cycle condition was optimized as: initial denaturation at 95 °C for 30 s, denaturation at 95 °C for 15 s, annealing at 60 °C for 30 s, elongation at 72 °C for 10 s for 40 cycles. At the end of the reaction, a melting curve analysis (65–105 °C) was carried out to check for the presence of primer dimers. The relative concentration of target genes was determined by cycle threshold (Ct) at which specific fluorescence became detectable. The Ct value was used for kinetic analysis and was proportional to the initial number of target copies in the sample. The qRT-PCR data was exported and processed using the ΔΔCT method.

### Immunoblot analysis

Sertoli cells were lysed in immunoprecipitation (IP) lysis buffer added with several protease inhibitors and phosphatase inhibitors. 50 *μ*g cell lysates were resolved for SDS-PAGE, primary antibodies were listed in Supplementary Table 2. Protein estimation was conducted on BioRad Model 680 Plate Reader with BioRad Dc Protein Assay Kit.

### Immunofluorescence analysis

4% Paraformaldehyde in PBS was used to fix Sertoli cells. Cells were then incubated with targeting primary antibodies at RT overnight at proper dilutions (Supplementary Table 1). FITC-488 or CY3-555 (Invitrogen) -conjugated secondary antibodies were used to visualize the signals. Images were taken by Olympus FSX100 fluorescence microscope and analyzed by Adobe Photoshop CS software for allowable image processing.

### TER measurement

TER measurement was conducted as described earlier. Briefly, Sertoli cells were cultured in Matrigel-coated bicameral units (in triplicates) at 1.2 × 10^6^ cells/cm^2^, the TER in each unit was read and recorded daily, the culture medium was replaced after TER measurement. On day 3, Sertoli cells were transfected by RNAi or treated with F5-peptide to investigate the effect of *abcb1a* and/or *abcb1b* silencing or F5-peptide on the integrity of Sertoli cell–TJ permeability barrier, respectively.

### Gene silencing by RNAi

After 3 days of culture, Sertoli cells were treated with scramble, *Abcb1a*-specific, *Abcb1b*-specific, or (*Abcb1a*-specific+*Abcb1b*-specific) siRNA duplexes with RiboJuice siRNA Transfection Reagent (Novagen; EMD Biosciences) as the transfection medium. To silence *Abcb1a*, a mixture of 75 nM *Abcb1a* (5′-GGCUUGCUGUUAUUACCCAtt-3′, s139475; Ambion) plus 75 nM scramble (Ambion) siRNA duplexes was used; To silence *Abcb1b*, a mixture of 75 nM *Abcb1b* (5′-GGCUUGCUGUAGUUACCCAtt-3′, s128200; Ambion) plus 75 nM scramble siRNA duplexes was used; To simultaneously silence *Abcb1a* and *Abcb1b*, a mixture of 75 nM *Abcb1a* and 75 nM *Abcb1b* siRNA duplexes was used; 150 nM scramble siRNA duplexes serve as non-targeting control. Following 24-h transfection, cells were feeded with fresh F12/DMEM and incubated for additional 24 or 48 h for subsequent experiments. siGLO Red Transfection Indicator (Dharmacon; Thermo Fisher Scientific) at a working concentration of 2 nM was cotransfected with siRNA duplexes to visualize the successful transfection in certain immunofluorescence experiments.

### Endocytosis assay

Endocytosis assay was conducted 3 days after RNAi transfection. Sertoli cells were pre-washed with cold PBS/CM (0.15 M NaCl, 10 mM NaH_2_PO_4_•H_2_O, 0.33 mM MgCl_2_, 0.9 mM CaCl_2_, pH=7.4,) twice, followed by 30-min biotinylation by using sulfo-NHS-SS-Biotin at 0.5 mg/ml (Pierce, Rockford, IL, USA) at 4 °C to biotinylate the cell surface proteins. NH_4_Cl at 50 mM was used to quench the free biotins at 4 °C for 15 min. Thereafter, cells were cultured for specific time points: 5, 15, 30, 60, 90 min at 35 °C. Stripping buffer (pH at 8.6, 50 mM MESNA, 100 mM Tris/HCl, 100 mM NaCl, and 2.5 mM CaCl_2_) was used to strip the uninternalized free biotins on cell surface at 4 °C for 30 min. Iodoacetamide at 5 mg/ml was then used to quench cells at 4 °C for 15 min. Finally the cultures were harvested in IP lysis buffer containing 0.2% SDS. Cell lysate with 30 min biotinylation but without stripping was used as total biotinylated surface protein, cell lysate without biotinylation served as negative control. 200 μg lysates plus 25 μl NeutrAvidin beads (Pierce) were co-incubated with at RT overnight to extract the biotinylated proteins. Beads were then cleaned with IP lysis buffer (containing 0.2% SDS) four times for a total 1.5 h, spinned down at 4000 × *g*, and subjected to SDS-PAGE.

### Recycling assay

Shortly, Sertoli cells on day 3 post-RNAi treatment were biotinylated on the surface proteins, followed by 2 h incubation in a 35 °C incubator to initiate and finish endocytosis. 30-min MESNA treatment at 50 mM (containing100 mM Tris/HCl, 100 mM NaCl, and 2.5 mM CaCl_2_, pH=8.6) was used to strip the remaining cell surface biotin, and iodoacetamide at 5 mg/ml was used in the quenching step for 15 min. Cells were then cultured for 0, 15, 30 and 60 min at 35 °C to initiate the protein recycling. Proteins that returned to cell surface were again stripped with MESNA and quenched with iodoacetamide as above described. Immunoblotting was then conducted to measure the decline on the levels of internalized proteins in the cytosol. In the meantime, we assess the reappearance of biotinylated protein to cell surface. Briefly, at selected time points after the initiation of protein recycling, cells were treated with 0.01% trypsin for 20 min to extract the recycled proteins on cell surface, followed by neutralizing trypsin with 1% soybean trypsin inhibitor. UltraLink Immobilized NeutrAvidin Plus beads (Pierce) was used to extract recycled proteins that were subsequently assessed by western blotting.

### Statistics

Each experiment was repeated at least three times using cultured Sertoli cells from different batches of SD rats. The data was analyzed by using GB-STAT software (version 7.0). Student’s *t*-test or one-way ANOVA coupled with a *post hoc* Tukey/Kramer procedure or two-tailed Dunnett’s test was employed to evaluate the significance between different treatment groups.
