# Supplementary material for: Abcb1a and Abcb1b genes function differentially in blood–testis barrier dynamics in the rat
Source: Cell Death Dis. 2017 Sep 7;8(9):e3038–. doi: 10.1038/cddis.2017.435 (PMC5636980; doi:10.1038/cddis.2017.435)
Supplement: Supplementary Material [file cddis2017435x1.docx]

***Abcb1a* and *Abcb1b* genes function differentially in blood-testis barrier dynamics in the rat**

Linlin Su^1, *^, Yan C. Cheng^2^, Will M. Lee^3^, Min Zhang^1^, Fangfang Yang^1^, Bin Zhao^1^, Daishu Han^4^, Yixun Liu^5^, Dahai Hu^1, *^

^1^Department of Burns and Cutaneous Surgery, Xijing Hospital, the Fourth Military Medical University, Xi’an, Shaanxi 710032, China.

^2^The Mary M. Wohlford Laboratory for Male Contraceptive Research, Population Council, Center for Biomedical Research, New York, NY 10065, USA.

^3^School of Biological Sciences, University of Hong Kong, Hong Kong SAR, China.

^4^Department of Cell Biology, Institute of Basic Medical Sciences, Chinese Academy of Medical Sciences, Peking Union Medical College, Beijing 100005, China.

^5^State Key Laboratory of Stem Cells and Reproductive Biology, Institute of Zoology, Chinese Academy of Sciences, Beijing 100101, China; University of Chinese Academy of Sciences, Beijing, 100049, China.

^*^Correspondence should be addressed to:

Linlin Su, [linlinsu@fmmu.edu.cn](mailto:linlinsu@fmmu.edu.cn); Dahai Hu, [hudhai@fmmu.edu.cn](mailto:hudhai@fmmu.edu.cn). Department of Burns and Cutaneous Surgery, Xijing Hospital, the Fourth Military Medical University, No.127 Changle West Road, Xi’an, 710032, China. Tel: +86-29-8477 5298, Fax: +86-29-8325 1734.

**
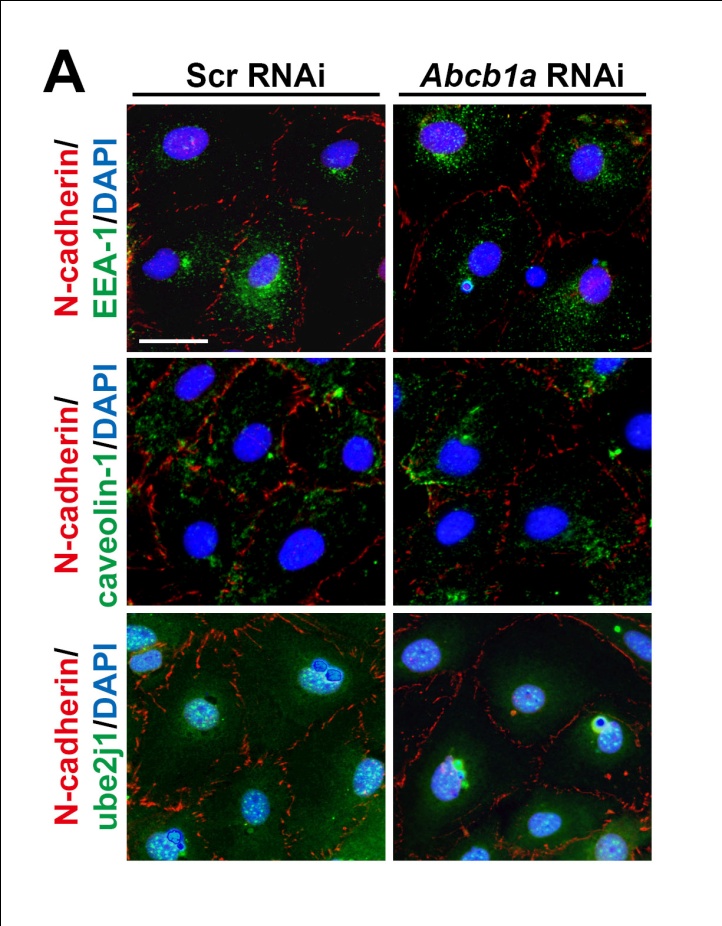
**

**Figure S1. Study to assess the effect of *Abcb1a* single knockdown on the endocytosis of basal ES protein N-cadherin and the endocytic vesicle-mediated protein trafficking in Sertoli cell epithelium.** (***A***) Cellular colocalization of EEA-1, caveolin-1, or ube2j1 with N-cadherin in Sertoli cells was examined by dual-labeled immunofluorescence analysis after *Abcb1a* knockdown. No change in the colocalization of N-cadherin with EEA-1, caveolin-1, or ube2j1 was observed after *Abcb1a* knockdown. DAPI (*blue*) was used to visualize nuclei, scale bar = 20 μm, which applies to all micrographs.

| **Gene** | **Primer sequence** | **Orientation** | **Position** | **Length (bp)** | **A.T.**  **(°C)** | **Cycle**  **no.** | **GenBank^®^**  **accession no.** |
| --- | --- | --- | --- | --- | --- | --- | --- |
| ***Abcb1a*** | 5’- CGCTCCCACTTATGA-3’ | sense | 273-287 | 96 | 60 | 40 | NM_133401.1 |
|  | 5’- GATGTTACGATGTCTGT-3’ | anti-sense | 352-368 |  |  |  |  |
| ***Abcb1b*** | 5’- AACCCTGCTTCCCCTCCTGA-3’ | sense | 215–234 | 119 | 60 | 40 | NM_012623.2 |
| ***GAPDH*** | 5’- GAAATCAACAGTACACAGACCG-3’  5’- GGCACAGTCAAGGCTGAGAATG-3’  5’- ATGGTGGTGAAGACGCCAGTA-3’ | anti-sense  sense  anti-sense | 312–333  241-262  363-383 | 143 | 60 | 40 | NM_017008.3 |

**Table S1.** Primers used for quantitative real-time PCR in this study.

A.T., annealing temperature.

**Table S2.** Summary of primary antibodies used in this study.

| **Target protein** | **Catalog#** | **Host** | **Vendor** | **Working Dilution** | |
| --- | --- | --- | --- | --- | --- |
|  |  |  |  | **IB** | **IF** |
| **P-glycoprotein** | sc-55510 | mouse | Santa Cruz Biotechnology | 1:200 | 1:50 |
| **MRP1** | sc-13960 | rabbit | Santa Cruz Biotechnology | 1:200 |  |
| **occludin** | 71-1500 | rabbit | Invitrogen | 1:250 | 1:50 |
| **JAM-A** | 36-1700 | rabbit | Invitrogen | 1:250 |  |
| **ZO-1**  **ZO-1-FITC** | 61-7300  33-9111 | rabbit  mouse | Invitrogen  Invitrogen | 1:250 | 1:50  1:50 |
| **N-cadherin** | sc-7939  33-3900 | rabbit  mouse | Santa Cruz Biotechnology | 1:200 | 1:50  1:50 |
| **β-catenin** | 71-2700 | rabbit | Invitrogen | 1:250 | 1:50 |
| **FAK** | 06-543 | rabbit | Millipore | 1:1000 |  |
| ***p*-FAK-Tyr^397^** | 44-625G | rabbit | Invitrogen | 1:1000 |  |
| ***p*-FAK-Tyr^407^** | 44650G | rabbit | Invitrogen | 1:1000 |  |
| **EEA-1** | 610457 | mouse | BD Biosciences | 1:2000 | 1:100 |
| **caveolin-1** | sc-53564 | mouse | Santa Cruz Biotechnology | 1:200 | 1:100 |
| **ube2j1** | WH0051465M1 | mouse | Sigma-Aldrich | 1:300 | 1:100 |
| **actin** | sc-1616 | goat | Santa Cruz Biotechnology | 1:200 |  |

IB, immunoblotting; IHC, immunohistochemistry; IF, immunofluorescence.
